# RETRACTION: Herbal Compound “Songyou Yin” Renders Hepatocellular Carcinoma Sensitive to Oxaliplatin through Inhibition of Stemness

**DOI:** 10.1155/ecam/9781942

**Published:** 2026-05-08

**Authors:** Evidence-Based Complementary and Alternative Medicine

RETRACTION: Q.‐A. Jia, Z.‐G. Ren, Y. Bu, et al., “Herbal Compound “Songyou Yin” Renders Hepatocellular Carcinoma Sensitive to Oxaliplatin through Inhibition of Stemness,” *Evidence-Based Complementary and Alternative Medicine* 2012, no. 1 (2012): 908601, https://doi.org/10.1155/2012/908601.

The above article, published online on 20 December 2012 in Wiley Online Library (https://wileyonlinelibrary.com), has been retracted by John Wiley & Sons Ltd. The retraction has been agreed upon following concerns raised by a third party. An investigation identified several duplications between images included in Figures 3c, 4, and 5 of this article and three other articles published by some of the same authors. In these instances, the duplicate images were used to represent different cell lines, tumors, or other biological contexts. A further duplication was identified between the C90, Oxa +SYY panel and the EpCAM, Oxa +SYY panel of Figure 5 in this article. The authors cooperated with the investigation and explained that the duplicated images resulted from figure mismanagement, noting that the articles containing the duplicates were published after this article. However, they did not provide data that met the publisher’s criteria for raw data. Due to the extent and nature of the concerns, the publisher considers the results and conclusions of this study insufficiently supported. The authors disagree with the retraction.

